# Editorial: Diagnostic accuracy of sepsis: clinical scores combination and serum biomarkers for rapid diagnosis and prognosis

**DOI:** 10.3389/fmed.2023.1252213

**Published:** 2023-09-26

**Authors:** Silvia Spoto, César Bustos, Silvia Angeletti

**Affiliations:** ^1^Diagnostic and Therapeutic Medicine Department, Fondazione Policlinico Universitario Campus Bio-Medico, Rome, Italy; ^2^Division of Infectious Diseases, Department of Internal Medicine, Clinica Universidad de los Andes, Santiago, Chile; ^3^Unit of Laboratory, Fondazione Policlinico Universitario Campus Bio-Medico, Rome, Italy; ^4^Research Unit of Clinical Laboratory Science, Department of Medicine and Surgery, Università Campus Bio-Medico di Roma, Rome, Italy

**Keywords:** sepsis, serum biomarkers, clinical scores, diagnostic accuracy of sepsis, prognosis

Sepsis is a life-threatening syndrome triggered by infection, accounting for 17% of intra-hospital mortality increasing up to 26% in case of septic shock with estimated costs of over 24 billion dollars per year (1). Early diagnosis and appropriate treatment can significantly reduce sepsis mortality (1). However, unfortunatelyat present no gold standard for sepsis diagnosis has been defined (1).

The combination of clinical scores and biomarkers increases the diagnostic and prognostic accuracy of sepsis, improving patient clinical management (2). Various clinical variables and tools are used for sepsis screening, such as vital signs, signs of infection, systemic inflammatory response syndrome (SIRS) criteria, quick Sequential Organ Failure Score (q-SOFA) or Sequential Organ Failure Assessment (SOFA) criteria, National Early Warning Score (NEWS), or Modified Early Warning Score (MEWS) (2).

The Research Topic was aimed to provide further evidence about the role of clinical scores and serum biomarkers combination in increasing accuracy and timely sepsis diagnosis. In this Research Topic, eight original research papers and one systematic review with meta-analysis were published. All of them brought evidence that sepsis diagnosis, a syndrome whose timely diagnosis has a significant impact patient's prognosis, can be facilitated by the combination of clinical data, in the form of scores, and the dosage of biomarkers. In recent years, a lot has been published about new biomarkers useful for this purpose, but there is still no signature sepsis biomarker, unlike other diseases, such as troponin in myocardial infarction. This happens because sepsis is a polymorphic syndrome with various stages of severity, potentially involving with alteration of different homeostatic mechanisms and consequent different biomarkers involvement. Some of these biomarkers are more specifically altered during sepsis, mainly those involved in inflammation such as C-reactive protein (CRP), Procalcitonin (PCT), and presepsin.

In this regard, Park et al. reported data from a retrospective cross-sectional study on 757 patients with culture-proven bacterial infections. Data showed that PCT and presepsin proved to be more promising biomarkers than CRP. Specifically, PCT showed the best performance in infection prediction, while presepsin yielded the best prognosis, proving their combination as a good tool to use in septic patients.

Within the Research Topic, two articles were included that evaluated biomarkers differently from those related to the inflammatory process, CRP, PCT, or presepsin, but related to organ damage or to innate or specific immunity activation. Zhao et al. in a retrospective cohort study on 456 patients with sepsis and sepsis-associated encephalopathy used the dosage of ammonium levels to correlate them to the prognosis. Authors reported a significant correlation of serum ammonia level with higher SOFA score and lactate but not with other prognostic factors such as hospital mortality or longer hospital stay, which, on the contrary, correlated significantly with Simplified Acute Physiology Score (SAPS II) and Charlson clinical score. These data confirmed that the combination of clinical scores and biomarkers can lead to rapid identification of patients at increased risk of death providing more targeted and effective monitoring.

Ma et al. performed a meta-analysis to investigate the accuracy of soluble-urokinase-type plasminogen activator receptor (SuPAR) in neonatal sepsis. This receptor is expressed on the membrane of immune cells, endothelial cells, and smooth muscle cells and is upregulated at sites of inflammation. It interacts and cooperates with many ligands and receptors, mainly integrins, to facilitate intracellular signaling, cell migration, cell adhesion, and tissue remodeling. SuPAR is released during inflammation or immune activation and although it is not disease-specific, its circulating levels reflect the severity and prognostic outcome of many infectious, inflammatory, and autoimmune disorders. The meta-analysis, while demonstrating the diagnostic potential of this biomarker, also highlighted that more high-quality studies are needed to confirm this data, given that the studies published so far are limited, there being only six.

Miyajima et al. analyzed parameters deriving from neutrophils cell population such as the Fluorescent light intensity (NE-SFL) and the Fluorescent light distribution Width (NE-WY) resulted in good indicators of sepsis compared to other biomarkers such as PCT, Interleukin 6 (IL-6), CRP and presepsin. In particular, it has been seen that NE-SFL and NE-WY are higher in patients with bacteremia and significantly associated with a high bacterial load as detected by the molecular PCR test. Furthermore, the levels of the two indicators significantly correlated with those of PCT and IL-6. The data of this study suggest that NE-SFL and NE-WY deriving from the analysis of cell population data may have a significant role in predicting severe bacterial infections.

An article about it was also included in the Research Topic a retrospective observational cohort study performed on 1,057 patients admitted to the Emergency Department after receiving antibiotic therapy for suspected sepsis (Sivayoham et al.). The aim of Sivayoham et al. was to risk-stratify sepsis patients for in-hospital mortality identifying the best risk-stratification tool for outcome at 180 days after admission. For risk stratification the following scores, Emergency Department suspected Sepsis (REDS) score, SOFA score, Red-flag sepsis criteria met, NICE high-risk criteria met, the NEWS2 score, and the SIRS criteria, were used. The results evidenced that 13.8% of patients died at hospital discharge and 27% died within 180 days, with overall survival of 74.4% at 180 days. Among the different scores the REDS and SOFA scores identified < 50% of the population as high-risk and all tools except the SIRS criteria, were relevant for outcome at 180 days. These data suggest that although all the risk-stratification tools were useful for outcome at 180 days, REDS and SOFA scores were superior to other tools, while SIRS criteria were useless for this purpose.

Regarding the indicators of prognosis and mortality, the study by Yang et al. (3) was focused on patients with acute kidney injury (AKI) and Central Venous pressure (CVP) for volume status assessment. The authors investigated the optimal time window to obtain CVP preventing adverse outcomes. They showed that delayed CVP time assessment was associated with a greater risk of in-hospital mortality, while prompt CVP monitoring contributed to shorter length of ICU stays and fewer days of norepinephrine use, as well as better fluid management.

Recently, great importance has been given to the application of artificial intelligence algorithms in the medical field especially to improve patients' diagnosis and treatment. It was therefore useful to include in the Research Topic two articles in which the use of artificial intelligence algorithms represented examples of application in the diagnosis and prognosis of patients with sepsis.

Wang et al. performed metabolomics profiling in septic patients compared to healthy subjects and applied five different machine learning (ML) algorithms to analyze the obtained data. The authors demonstrated that the occurrence of sepsis determines metabolite dysregulation, especially of mannose-6-phosphate and sphinganine, which are positively correlated with biomarkers such as PCT, leukocyte count, CRP, and Interleukin-6. These data suggested how ML could represent a useful approach for precision medicine delivery.

ML approach was used also by Cheng et al. for sepsis in-hospital mortality prediction within 48 h from symptoms onset. These authors used dynamic changes in the patient's vital signs such as systolic and diastolic blood pressures, heart and respiratory rates, and body temperature, concluding that machine learning models were useful for mortality prediction within 6 to 48 h from admission.

Two studies were carried out in patients affected by COVID-19 were also included. The first by Al-Shudifat et al. (4) investigated the correlation between lung computed tomography (CT) data and demographics or vital signs findings. This cross-sectional study revealed that several factors could represent predictors for outcome and lung changes, such as age above 60 years old, the presence of dry or productive cough, and more than three antibiotic prescriptions. The second article was by Rivera-Fernandez et al. who performed a multicenter prospective cohort study investigating the role of PCT measurement on mortality in patients with COVID-19 and respiratory involvement. These authors showed that PCT elevation was observed in several measurements and significantly correlated to mortality. Moreover, PCT was high in more than 50% of non-survivors until the final day before death. The authors concluded that the serial assessment of procalcitonin in these patients could be useful for death risk stratification.

We hope that this Research Topic provides a stimulus and an upgrade to the scientific community to promote an increasingly integrated line of research between clinic and laboratory. This could improve early and accurate diagnosis of serious and fatal diseases such as sepsis. This approach proves useful also in new infections, as COVID-19 has taught us.

## Author contributions

SS and SA drafted the manuscript. CB, SS, and SA critically revised the manuscript for important intellectual content. All authors contributed to the article and approved the submitted version.
